# Integrated Cranio-Cervical Organization in Adults: Association Between Morphocranial Pattern and Segmental Cervical Alignment After Orthodontic Treatment

**DOI:** 10.3390/jcm15166475

**Published:** 2026-08-21

**Authors:** Marta Serra Alierta, Meritxell Sánchez-Molins, Aroa Casado Rodriguez, Maria Cristina Manzanares Cespedes, Juan Gonzalo Olivieri

**Affiliations:** Department of Odontoestomatology, Faculty of Medicine and Health Sciences, Universitat de Barcelona (UB), Campus Bellvitge, 08907 L’Hospitalet de Llobregat, Barcelona, Spain

**Keywords:** cranial base, cervical vertebrae, cervical organization, orthodontics, adult, cephalometry, morphocranial pattern, cranio-cervical posture

## Abstract

**Objective:** This study aimed to examine the association between the study-specific Cl-Na/Cl-Ba cranial-base angle and segmental cervical organization in adults, considering both inclination and sagittal spatial position, and to explore whether longitudinal changes in cervical inclination differed among morphocranial groups over the observed orthodontic treatment interval. **Materials and Methods:** Pre-treatment and post-treatment lateral cephalograms from 297 adult orthodontic patients obtained in natural head position were analyzed. Cranial-base morphology was operationally classified using the Cl-Na/Cl-Ba angle into brachycephalic (<115°), mesocephalic (115–124°), and dolichocephalic (>124°) groups. Segmental cervical inclination (C2–C5) and horizontal and vertical vertebral coordinates were recorded. Intergroup differences were assessed using Kruskal–Wallis tests with Dunn–Holm post hoc comparisons and effect-size estimates. Associations between the continuous cranial-base angle and cervical variables were evaluated using Spearman correlations. Longitudinal changes in cervical inclination were examined using linear mixed-effects models adjusted for sex, age, cervical segment, and repeated measurements. **Results:** The continuous cranial-base angle was inversely correlated with all segmental inclination variables (ρ = −0.213 to −0.333; *p* ≤ 0.002). Spatial coordinates showed larger associations, particularly for vertical positioning (C2 Y: ρ = −0.653; *p* < 0.001). Morphocranial groups differed in segmental inclination and spatial coordinates, with the largest effect sizes observed for upper cervical vertical variables. In the mixed-effects model, time was significant (*p* = 0.014), and morphocranial pattern remained independently associated with cervical inclination (*p* < 0.001). No significant time × morphocranial-pattern interaction was observed (*p* = 0.184). **Conclusions:** In this retrospective adult orthodontic sample, the study-specific cranial-base angle was associated with segmental cervical inclination and sagittal spatial coordinates, with the largest associations observed in the upper cervical region. The longitudinal model provided no evidence of differential longitudinal changes in cervical inclination among morphocranial groups over the observed interval. These findings are observational and do not establish a causal cranio-cervical mechanism or a treatment-specific effect. Segmental angular and spatial variables may be complementary descriptive measures when interpreting cranio-cervical cephalograms.

## 1. Introduction

The relationship between craniofacial morphology and cervical posture has long been considered within the broader framework of cranio-cervical organization. Classical descriptions of this complex emphasize the anatomical and functional interdependence among the cranial base, mandible, head position, and cervical spine [1,2,3]. This relationship is clinically relevant in orthodontics because lateral cephalograms routinely capture cranial and upper cervical structures; however, their interpretation requires caution. The available literature generally reports low-to-moderate and heterogeneous associations between craniofacial morphology and cervical posture, rather than a deterministic relationship between either system [4,5,6].

Part of this heterogeneity may arise from the way cervical organization has been assessed. Many cephalometric studies rely on global angular variables, which may not distinguish the behavior of individual cervical segments [7,8,9,10,11,12,13]. In addition, segmental inclination and vertebral spatial position represent complementary dimensions. Two individuals may have similar angular measurements while differing in the anteroposterior or vertical location of the cervical chain. A segment-specific approach that combines angular and spatial variables may therefore provide a more complete description of cranio-cervical organization and may help identify whether associations vary along the cranio-caudal axis.

The available studies of orthodontic treatment have reported modest or variable changes in cranio-cervical variables [14,15]. Longitudinal adult data are therefore needed to distinguish changes observed over the treatment interval from pre-existing patterns of cranio-cervical organization. Such data cannot establish a treatment effect without an untreated comparison group, but they can explore whether longitudinal changes in cervical inclination differ among morphocranial groups over time.

A further methodological issue is terminology. In the present study, morphocranial pattern refers exclusively to an operational classification based on the study-specific Cl-Na/Cl-Ba cranial-base angle. It is not intended to be synonymous with conventional vertical facial-growth patterns. The rationale for retaining this study-specific construction and the limits of comparing it with conventional cranial-base measurements are detailed in the Section 2.

Accordingly, the primary objective of this retrospective longitudinal study was to examine the association between the study-specific cranial-base angle and segmental cervical organization in adults, considering both segmental inclination and the sagittal spatial position of C2–C5. The secondary objective was to explore whether longitudinal changes in cervical inclination differed among morphocranial groups over the observed orthodontic treatment interval. The study was designed to test two a priori hypotheses: first, that the study-specific cranial-base angle would be associated with both angular and spatial cervical variables, and second, that the magnitude of these associations would be greater in the upper cervical region than at C5.

## 2. Materials and Methods

### 2.1. Study Design

A retrospective longitudinal observational study was conducted following the STROBE (Strengthening the Reporting of Observational Studies in Epidemiology) guidelines. The study was designed to evaluate the association between cranial-base morphology and segmental cervical organization in adult orthodontic patients, as well as the longitudinal stability of these associations after orthodontic treatment. The study protocol was approved by the Ethics Committee of the Hospital Odontològic Universitat de Barcelona (HOUB) (Ref: 23/2020; date: 7 July 2020).

### 2.2. Clinical Setting and Sample Selection

Patients were recruited from the institutional database of the HOUB, including subjects who completed orthodontic treatment between 2010 and 2020. All treatments were performed by postgraduate orthodontic students under the supervision of experienced clinical orthodontists, following standardized diagnostic and therapeutic protocols established by the institution.

Clinical and radiographic records were consecutively reviewed to determine patient eligibility according to predefined selection criteria.

### 2.3. Inclusion Criteria

- Caucasian patients older than 18 years.
- Availability of pre-treatment and post-treatment lateral cephalograms obtained in natural head position (NHP).
- Adequate visualization of the occipital base and cervical spine for the planned cephalometric analysis.
- Clearly identifiable osseous landmarks free of radiographic artifacts.
- Absence of previous craniofacial surgery or cranio-cervical trauma.
- Presence of at least 16 teeth, including a minimum of four posterior teeth, verified by pre-treatment panoramic radiography.
- Orthodontic treatment performed using fixed appliances and/or aligners.

### 2.4. Exclusion Criteria

- Craniofacial malformations or facial paralysis.
- Previous orthognathic surgery or cranio-cervical trauma.
- Radiographs unsuitable for cephalometric analysis.
- Diagnosis of severe respiratory disorders.

### 2.5. Sample Size Calculation

Sample size estimation was performed using GPower software (version 3.1.9.7; Heinrich Heine University Düsseldorf, Düsseldorf, Germany). Assuming a significance level of α = 0.05, a statistical power of 80%, a small-to-moderate effect size (Cohen’s *f* = 0.20), and a repeated-measures correlation of 0.50, a minimum sample size of approximately 250 participants was considered sufficient to detect differences in cervical organization according to morphocranial pattern in longitudinal analyses. The final sample of 297 patients exceeded this requirement, providing adequate statistical power for the repeated-measures models and group comparisons.

### 2.6. Radiographic Acquisition

Lateral cephalograms were obtained in natural head position using a Planmeca ProMax unit (Planmeca USA, Inc., Roselle, IL, USA) with the following parameters: 66 kV, 10.0 mA, exposure time of 10.5 s, 2.5 mm Al filtration, and 10% magnification.

Natural head position was obtained using a standardized protocol in which patients remained seated with their gaze directed toward a point located at eye level, without head support and with relaxed cervical musculature. This protocol was systematically applied during both pre-treatment and post-treatment acquisitions by operators specifically trained in orthodontic radiology in order to minimize postural variability related to radiographic acquisition.

### 2.7. Cephalometric Analysis

Cephalometric analysis was performed using Nemoceph software version 2019 (Nemotec, Madrid, Spain). Because the present study incorporated an original segmental analysis based on vertebral inclination and spatial localization according to cervical level, a specific cephalometric protocol was developed in collaboration with the Nemoceph programming team.

Radiographs were digitized and calibrated before cephalometric tracing. All cephalometric tracings were performed by a single calibrated examiner (M.S.A.) using the same software and standardized tracing protocol. Each cephalometric variable was measured independently on three occasions, and the arithmetic mean of the three determinations was used as the definitive value in the statistical analyses. During the initial examiner-calibration phase, intraobserver reproducibility was formally evaluated and showed acceptable consistency with low measurement error; on this basis, the three-measurement averaging protocol was adopted for the study. As this reliability assessment was conducted during the original data-processing phase and only the definitive averaged dataset was retained, the numerical ICC outputs and the individual repeated determinations are no longer retrievable and therefore cannot be reported retrospectively.

**Figure 1:**
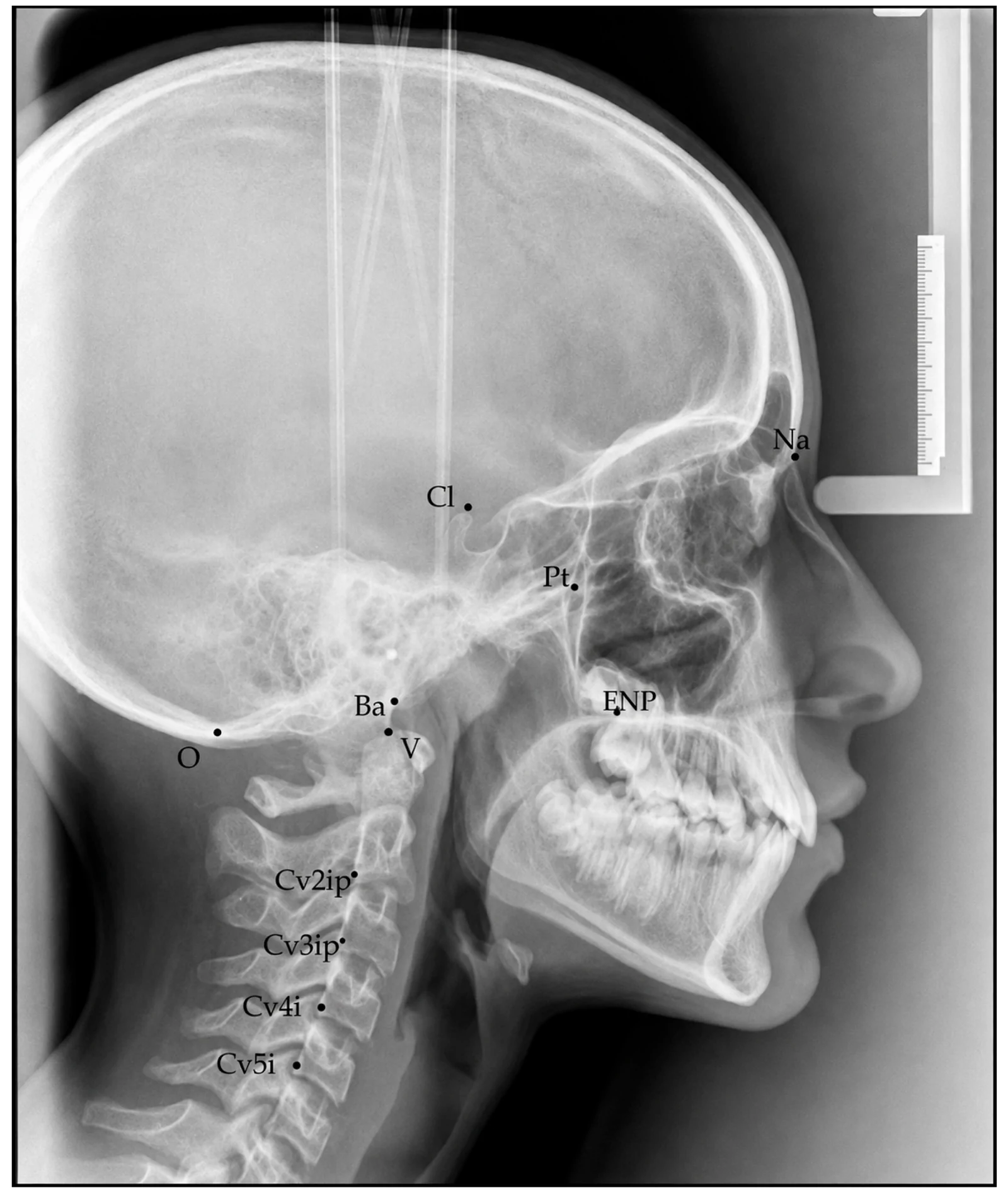
Cephalometric landmarks and cervical reference points. The figure identifies the cranial landmarks (Cl, Na, Ba, O, ENP, and Pt), the odontoid vertex (V), and the posteroinferior points of C2–C5 (Cv2ip–Cv5ip) used for angular and spatial measurements.

**Figure 2:**
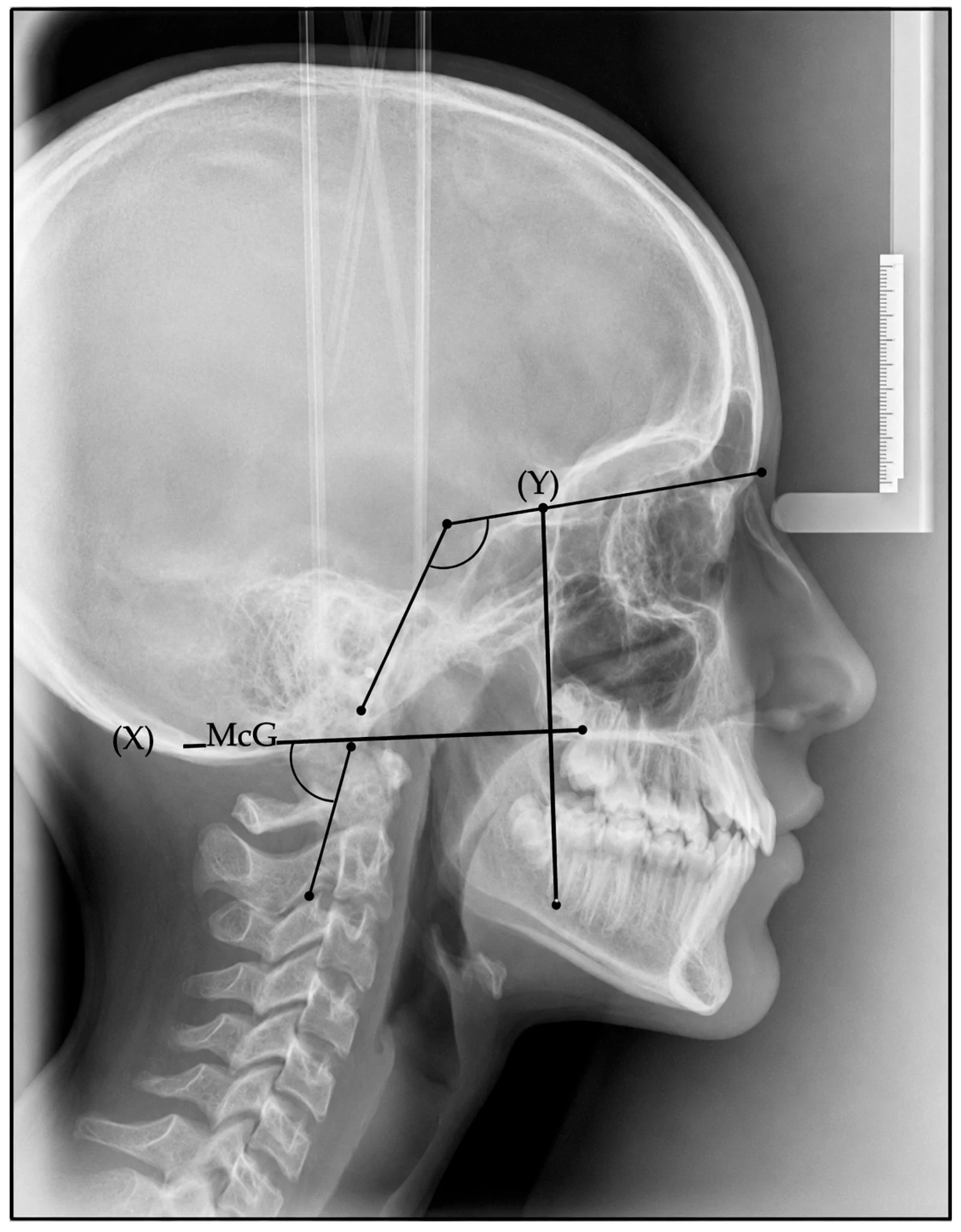
Cranial base and cervical reference planes used for morphocranial and segmental cervical analysis. The Cl-Na and Cl-Ba lines define the study-specific cranial base angle; McG is the McGregor plane. OPTm, CVTm, CVT, and EVTm represent the segment-specific cervical tangents for C2–C5, respectively.

The cephalometric landmarks, reference lines, and reference planes used in the study are presented in Table 1 and Table 2 and illustrated in Figure 1 and Figure 2.

The Cl landmark and the Cl-Na/Cl-Ba angle were retained because they formed part of the original institutional tracing protocol and the available longitudinal dataset. This construction differs from the conventional N-S-Ba or N-S-Ar cranial base angles commonly used in orthodontic cephalometry [16]. Accordingly, results from the present angle should not be assumed to be numerically interchangeable with studies using N-S-Ba, N-S-Ar, or DS-based constructions.

### 2.8. Study Variables

#### 2.8.1. Demographic Variables

The following demographic variables were recorded:

- Sex (male/female).
- Age, categorized into three groups: 18–25 years, 26–45 years, and >45 years.

#### 2.8.2. Craniofacial Variables

Cranial base morphology was determined using the Cl-Na/Cl-Ba angle, classifying subjects into three morphocranial patterns:

- Brachycephalic (<115°).
- Mesocephalic (115–124°).
- Dolichocephalic (>124°).

The three categories were used as study-specific operational groups based on the prespecified cut-offs of the institutional protocol. They should not be interpreted as externally validated normative thresholds or as direct equivalents of brachyfacial, mesofacial, and dolichofacial vertical growth patterns. The use of Cl, rather than Sella, was selected for the present cephalometric protocol because it provided a clearly identifiable anatomical reference in the study radiographs. The Cl-Na/Cl-Ba angle was also analyzed as a continuous variable to reduce dependence on categorical cut-offs. Further studies should assess the reproducibility and external validity of this construction and its relationship with conventional cranial-base measurements, including N-S-Ba.

#### 2.8.3. Cervical Variables

Cervical organization was evaluated using both angular and spatial segmental variables corresponding to cervical levels C2, C3, C4, and C5. Spatial analysis included both the odontoid vertex of C2 (V) and the posteroinferior vertebral points (Cv2ip–Cv5ip), thereby allowing independent assessment of upper and middle cervical organization (Figure 1).

#### 2.8.4. Segmental Inclination Variables

Segmental cervical inclination was evaluated using the following angular variables:

- C2 (OPTm/McG).
- C3 (CVTm/McG).
- C4 (CVT/McG).
- C5 (EVTm/McG).

OPTm denotes the modified odontoid process tangent used to represent C2 inclination; CVTm denotes the modified cervical vertebral tangent used for the C3 segment; CVT denotes the cervical vertebral tangent used for C4; and EVTm denotes the modified extended vertebral tangent used for C5. Each tangent was measured relative to the McGregor plane (McG), as illustrated in Figure 2. The suffix “m” indicates the study-specific modified construction implemented in the Nemoceph protocol.

#### 2.8.5. Vertebral Spatial Position

The spatial position of C2–C5 was recorded using horizontal (X) and vertical (Y) coordinates, with the McGregor plane as the horizontal reference and its perpendicular as the vertical reference.

The inclusion of spatial coordinates in addition to segmental inclination was based on the fact that both dimensions provide complementary, non-redundant information. Whereas inclination describes the relative angular orientation of each vertebral segment, spatial coordinates characterize its location in the sagittal plane.

All available radiographs were included in the analyses. When C5 landmarks were not identifiable, the corresponding C5-related measurement was treated as unavailable. C5-related analyses were based on 218 patients with an identifiable C5 landmark in both the pre-treatment and post-treatment radiographs.

The complete definitions of the cephalometric variables analyzed are presented in Table 3.

### 2.9. Statistical Analysis

Statistical analyses were performed using R software version 4.6.0 (R Foundation for Statistical Computing, Vienna, Austria).

Descriptive statistics were calculated for all cephalometric and cervical variables. The normality of distribution was evaluated using the Shapiro–Wilk test, complemented by skewness and kurtosis analyses. According to normality results, comparisons among morphocranial patterns were performed using the Kruskal–Wallis test. Post hoc comparisons were conducted using Dunn’s test with Holm correction for multiple comparisons. Effect size was estimated using Kruskal–Wallis eta squared (η^2^[H]), interpreted according to conventional criteria as small, moderate, or large.

Associations between continuous cranial morphology and cervical variables were evaluated using Spearman rank correlations (ρ), with correlation coefficients and corresponding *p*-values reported.

To analyze the longitudinal stability of cervical inclination between pre- and post-treatment records, a linear mixed-effects model was fitted using the lmer function, with time (pre/post), morphocranial pattern, sex, age group, cervical segment, and the time × morphocranial pattern interaction included as fixed effects. The individual subject identifier was incorporated as a random intercept to account for within-subject dependency across repeated measurements. Robust standard errors were additionally estimated using CR2 covariance matrices to verify the stability of the model coefficients.

A sensitivity analysis was conducted by excluding variables corresponding to the lower cervical segment (C5) to evaluate the consistency of findings across the upper and middle cervical segments.

Statistical significance was set at *p* < 0.05 for all analyses.

## 3. Results

### 3.1. Sample Characteristics

The sample consisted of 297 adult subjects classified according to morphocranial pattern into brachycephalic (*n* = 60; 20.2%), mesocephalic (*n* = 175; 58.9%), and dolichocephalic individuals (*n* = 62; 20.9%) (Table 4).

Descriptive analyses demonstrated adequate distributional stability across the majority of variables analyzed. Angular cervical variables showed approximately symmetric distributions with acceptable kurtosis values, whereas spatial coordinates demonstrated greater variability, particularly in inferior cervical levels. The vertical position of the odontoid vertex (V Y) demonstrated a stable distribution, with a mean of 4.78 ± 4.42 and normality consistent with a Gaussian distribution (Shapiro–Wilk test: *p* = 0.974).

**Figure 3:**
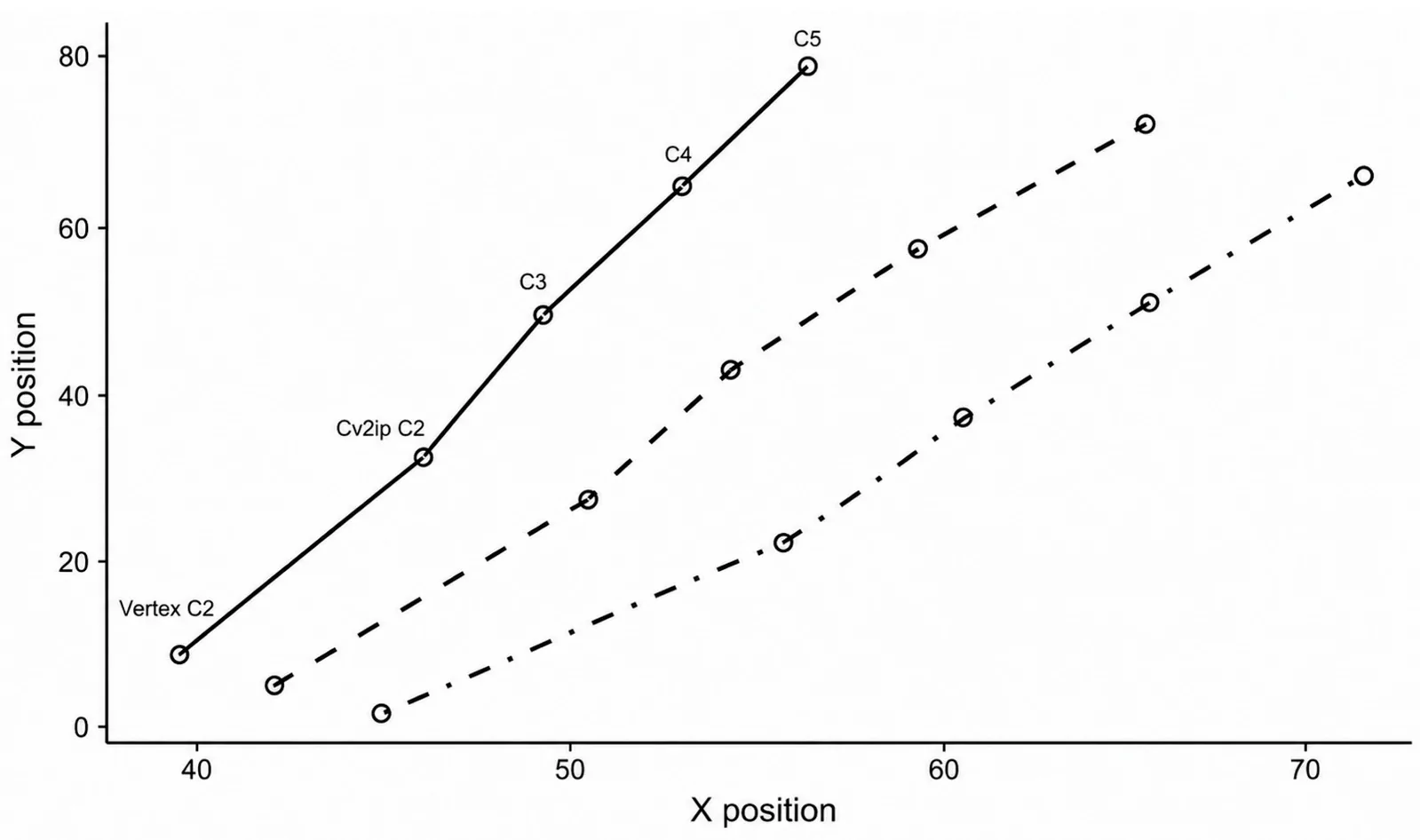
Cervical spatial organization according to morphocranial group. Horizontal (X) and vertical (Y) coordinates of the odontoid vertex (V) and the posteroinferior points of C2–C5 (Cv2ip–Cv5ip) are expressed relative to the McGregor plane and its perpendicular reference line.

**Figure 4:**
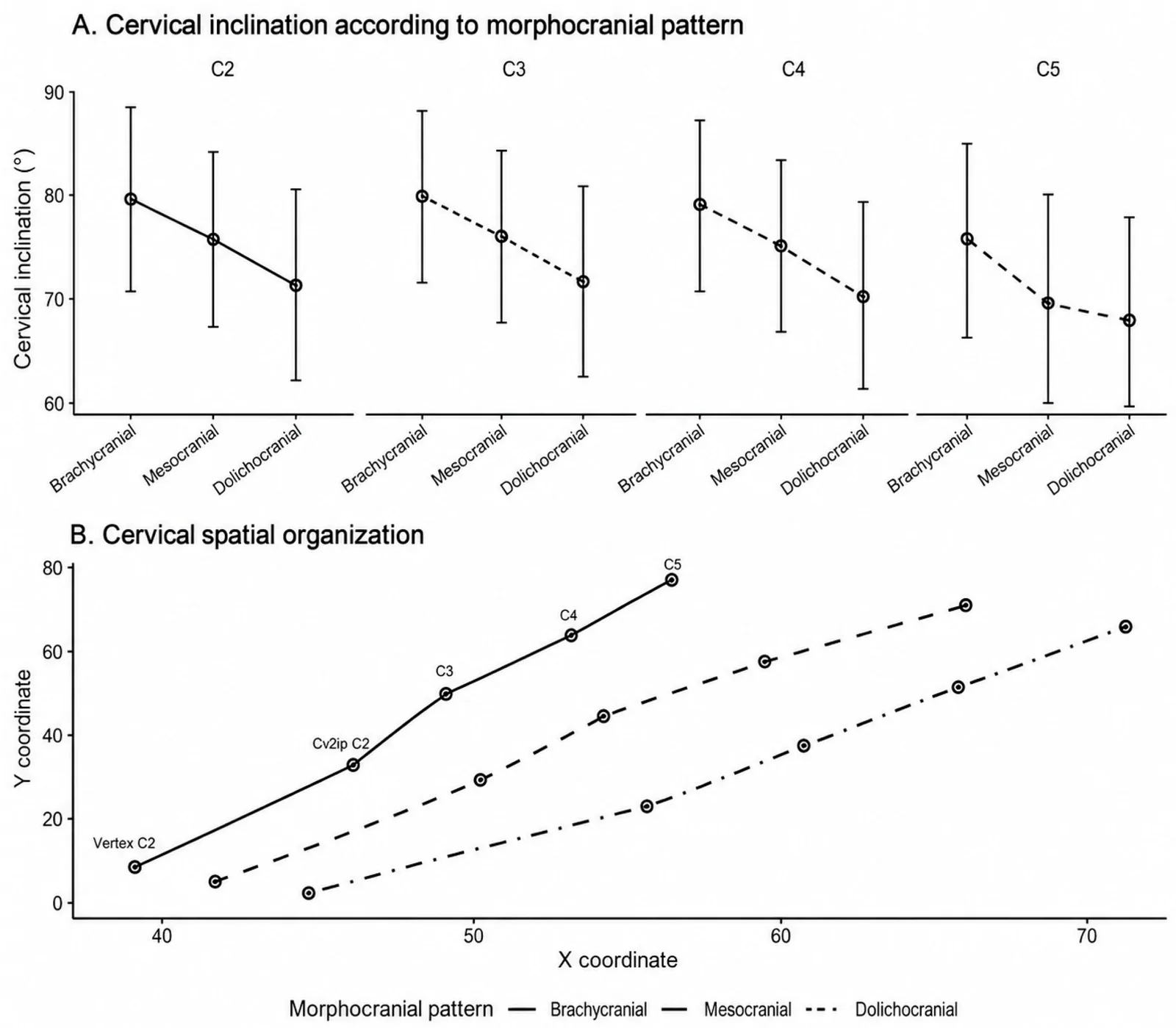
Segmental cervical inclination according to morphocranial group. Lines represent the mean inclination values for C2 (OPTm/McG), C3 (CVTm/McG), C4 (CVT/McG), and C5 (EVTm/McG).

Angular and spatial cervical variables differed significantly across morphocranial groups. Brachycephalic participants showed higher mean angular values and lower horizontal coordinate values than dolichocephalic participants, while vertical coordinate values decreased progressively across groups (Figure 3 and Figure 4).

### 3.2. Association Between the Continuous Cranial-Base Angle and Cervical Variables

Spearman correlation analyses showed statistically significant associations between the continuous Cl-Na/Cl-Ba cranial-base angle and all cervical variables evaluated (Table 5; Figure 3 and Figure 4).

All segmental inclination variables were inversely correlated with the cranial-base angle: C2 (ρ = −0.323, *p* < 0.001), C3 (ρ = −0.316, *p* < 0.001), C4 (ρ = −0.333, *p* < 0.001), and C5 (ρ = −0.213, *p* = 0.002). Among the angular variables, the largest absolute correlation coefficient was observed for C4.

Spatial coordinates also showed statistically significant associations with the cranial-base angle. Horizontal coordinates were positively correlated, with coefficients ranging from ρ = 0.408 for C5 X to ρ = 0.501 for C2 X (all *p* < 0.001). Vertical coordinates were inversely correlated, with coefficients ranging from ρ = −0.522 for C5 Y to ρ = −0.653 for C2 Y (all *p* < 0.001). The remaining vertical-coordinate correlations were ρ = −0.641 for V Y, ρ = −0.617 for C3 Y, and ρ = −0.583 for C4 Y (all *p* < 0.001).

The largest absolute correlation coefficients were observed for the vertical coordinates of V, C2, and C3. For both horizontal and vertical coordinates, the absolute magnitude of the correlation coefficients decreased from C2 to C5.

### 3.3. Segmental Cervical Inclination According to Morphocranial Pattern

Descriptive analyses demonstrated a progressive reduction in cervical inclination from brachycephalic to dolichocephalic subjects across all vertebral levels analyzed (Table 6; Figure 4).

For C2 inclination, mean values were 78.6 ± 8.2° in brachycephalic subjects, 74.9 ± 7.7° in mesocephalic subjects, and 70.5 ± 8.6° in dolichocephalic subjects. Similar progressive trends were identified for C3 inclination (78.8 ± 7.8°, 75.1 ± 7.7°, and 70.9 ± 8.5°, respectively) and C4 inclination (78.0 ± 7.7°, 74.2 ± 7.6°, and 69.7 ± 8.1°, respectively). Although C5 inclination followed the same global tendency, intergroup differences were less pronounced (74.7 ± 8.6°, 69.1 ± 9.2°, and 67.7 ± 8.9°, respectively).

Kruskal–Wallis analyses confirmed statistically significant differences between morphocranial groups for all segmental inclination variables (Table 7). Effect sizes ranged from small to moderate, with the highest values observed for C4 inclination (η^2^[H] = 0.095), followed by C2 inclination (η^2^[H] = 0.091) and C3 inclination (η^2^[H] = 0.086). C5 inclination presented the smallest effect size (η^2^[H] = 0.050).

Post hoc Dunn–Holm comparisons demonstrated significant differences between all morphocranial groups for C2, C3, and C4 inclination variables (all adjusted *p* < 0.01) (Table 8). In contrast, C5 inclination did not differ significantly between mesocephalic and dolichocephalic subjects (adjusted *p* = 0.362).

### 3.4. Spatial Cervical Organization According to Morphocranial Pattern

Spatial cervical coordinates differed across morphocranial groups (Table 9; Figure 3). Brachycephalic individuals showed lower horizontal coordinate values and higher vertical coordinate values than dolichocephalic individuals.

Across all cervical levels, mean horizontal coordinates increased from brachycephalic to mesocephalic and dolichocephalic subjects, whereas mean vertical coordinates decreased across the same groups (Table 9).

**Figure 5:**
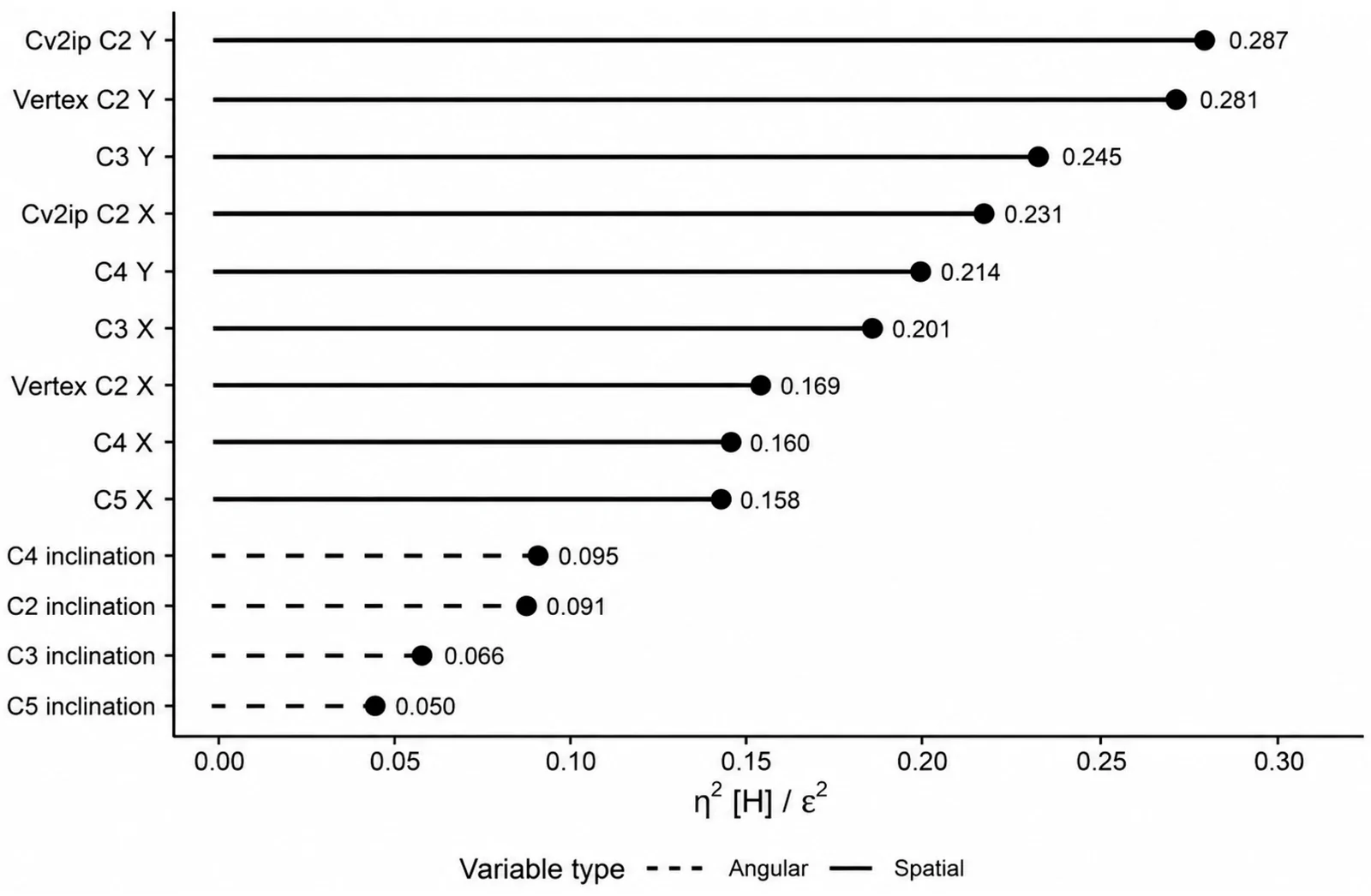
Effect sizes for angular and spatial cervical variables across morphocranial groups. Bars represent the Kruskal–Wallis effect-size estimates (η^2^(H) or ε^2^, as applicable) for segmental inclination variables (C2–C5) and spatial coordinate variables (V X, V Y, and C2–C5 X and Y).

Spatial variables showed larger effect sizes than angular variables. The largest effect sizes were observed for C2 Y (η^2^[H] = 0.287), V Y (ε^2^ = 0.281), C3 Y (η^2^[H] = 0.245), and C2 X (η^2^[H] = 0.231) (Figure 5).

Post hoc Dunn–Holm analyses confirmed significant differences between morphocranial groups for most spatial coordinates, particularly in the upper and middle cervical segments (Table 8). The most consistent pairwise differences were observed between brachycephalic and dolichocephalic subjects.

### 3.5. Vertical Coordinate Correlations

**Figure 6:**
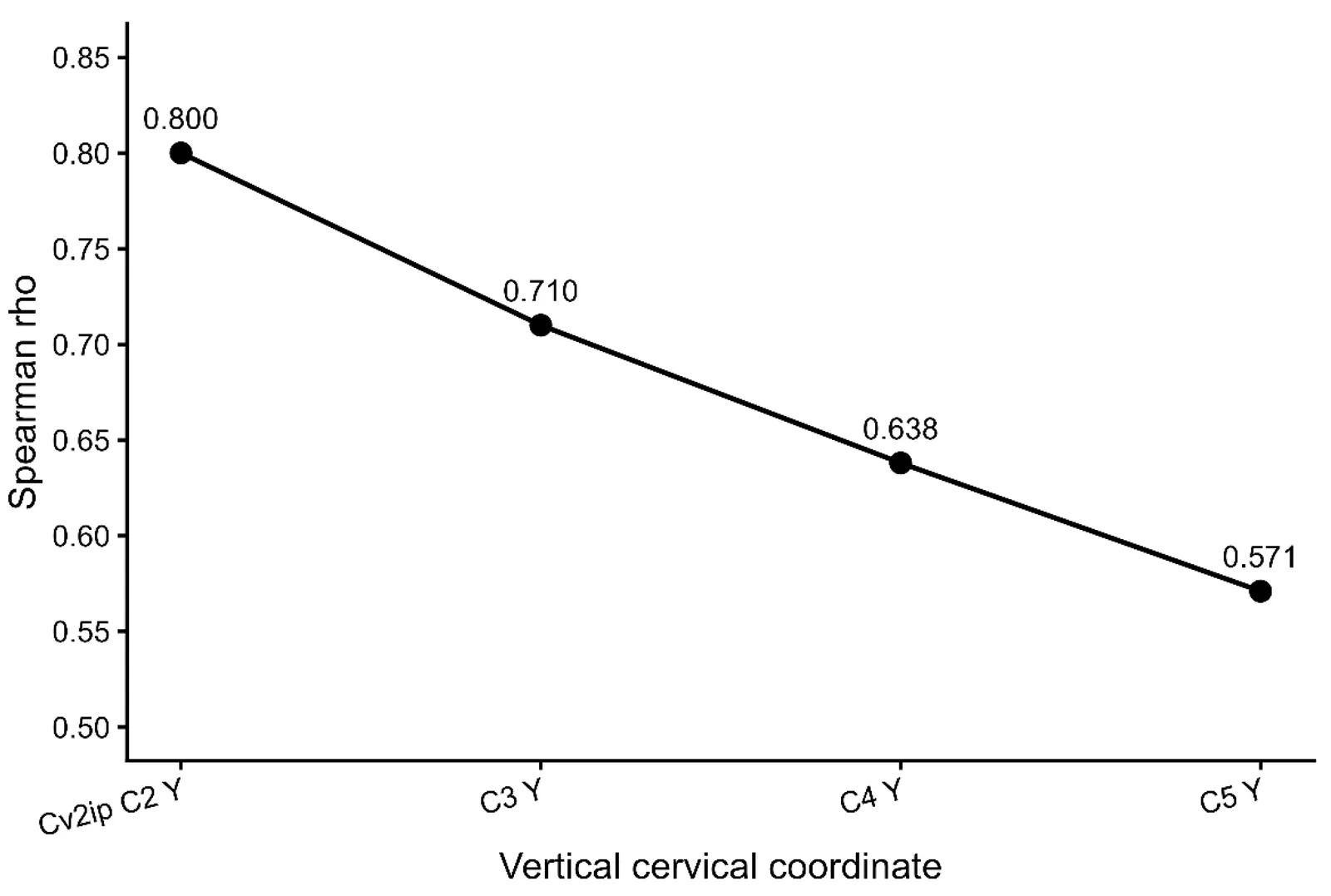
Correlations between the vertical coordinate of the odontoid vertex (V Y) and the vertical coordinates of C2–C5.

Correlations between the vertical coordinate of the odontoid vertex (V Y) and the remaining cervical vertical coordinates are presented in Table 10 and Figure 6. Positive correlations were observed between V Y and C2 Y (ρ = 0.800, *p* < 0.001), C3 Y (ρ = 0.710, *p* < 0.001), C4 Y (ρ = 0.638, *p* < 0.001), and C5 Y (ρ = 0.571, *p* < 0.001). The correlation coefficients decreased from C2 Y to C5 Y.

### 3.6. Longitudinal Analysis of Cervical Inclination

Linear mixed-effects analyses showed a statistically significant effect of time between the pre-treatment and post-treatment measurements (*p* = 0.014). The morphocranial pattern remained independently associated with cervical inclination after adjustment for age, sex, cervical segment, and repeated measurements (*p* < 0.001).

No significant time × morphocranial-pattern interaction was observed (*p* = 0.184). The model provided no evidence of differential longitudinal changes in cervical inclination among the brachycephalic, mesocephalic, and dolichocephalic groups over the observed interval.

## 4. Discussion

This retrospective longitudinal study examined the association between a study-specific cranial-base angle and segmental cervical organization in adults. The first hypothesis was supported: the Cl-Na/Cl-Ba angle was associated with both segmental inclination and sagittal spatial coordinates. The second hypothesis was also supported, as the largest associations were observed for the vertical coordinates of V, C2, and C3, and the magnitude of the associations decreased toward C5. With respect to the secondary longitudinal objective, no significant time × morphocranial-pattern interaction was observed; thus, the model provided no evidence of differential longitudinal changes in cervical inclination among the three morphocranial groups over the observed treatment interval. A summary of the principal findings, the interpretation supported by the data, and the corresponding cautions is provided in Table 11.

The spatial findings add information that is not captured by angular measurements alone. Although segmental inclinations were associated with the cranial-base angle, the largest effect sizes and correlation coefficients were observed for the vertical spatial coordinates. This result indicates that, within the present cephalometric protocol, vertebral location and segmental inclination describe complementary aspects of cervical organization. It does not establish cervical lordosis, kyphosis, or straightening, because global cervical curvature was not assessed. Previous studies and reviews have reported low-to-moderate and heterogeneous associations between craniofacial morphology and cervical posture [4,5,6,9,10,11,12,13]. The present findings extend that literature by evaluating both angular and spatial variables at individual cervical levels in an adult sample.

The reduction in association magnitude from the upper to the lower cervical levels is compatible with the closer anatomical and functional relationship of the upper cervical region to cranial-base and head-position variables [1,2,3,17,18]. One plausible interpretation is that cervical organization reflects coordinated variation in vertebral position and inclination rather than isolated movement of a single segment. Head balance, upper cervical muscular forces, and soft-tissue relationships may contribute to this coordination [3,17,18,19]. However, these mechanisms were not measured in the present study. In addition, the coordinates were defined relative to the McGregor plane and its perpendicular reference line; therefore, the observed spatial pattern may reflect both vertebral position and the geometric relation of the cranial-base reference system to the cervical structures. The data consequently support an association between the measured variables, not a specific biomechanical mechanism.

The longitudinal findings should be interpreted with similar caution. A significant time effect was identified between the pre-treatment and post-treatment measurements, whereas the non-significant time × morphocranial-pattern interaction provided no evidence of differential longitudinal changes in cervical inclination among the groups. This result suggests that the between-group pattern observed at baseline was not differentially modified over the observed interval. It does not demonstrate that orthodontic treatment had no effect on cervical posture. The study had no untreated comparison group, and treatment mechanics and duration were heterogeneous; therefore, temporal changes cannot be attributed specifically to orthodontic treatment. This cautious interpretation is particularly important because the available treatment-related literature is limited and includes populations that differ from the present adult orthodontic sample [14,15].

The combined assessment of segmental inclination, spatial coordinates, and repeated observations is a strength of the study. It allowed level-specific associations to be examined rather than relying only on global cervical measurements. In clinical practice, these variables may be useful as complementary descriptive measures when interpreting cranio-cervical cephalograms. They should not be used as diagnostic thresholds, treatment targets, or evidence of a causal cranio-cervical relationship without external validation and prospective controlled studies.

Several methodological constraints should frame the interpretation of these findings. The retrospective observational design precludes causal inference. The Cl-Na/Cl-Ba angle and its categorical cut-offs were defined within the institutional protocol. Although Cl was selected as a clearly identifiable anatomical reference for the present cephalometric analysis, the external validity of this construction and its comparability with conventional cranial-base measures, including N-S-Ba, require further study. Although all tracings were performed by a single calibrated examiner and each variable was measured three times, the numerical reliability outputs and individual repeated determinations were not retained and therefore cannot be reported retrospectively. Two-dimensional cephalograms cannot assess axial rotation, transverse asymmetry, or three-dimensional cervical alignment, and natural head position does not eliminate short-term postural variability. Because C5 landmarks were not identifiable in all radiographs, C5-related analyses were based on fewer observations than analyses of the upper cervical levels and should be interpreted accordingly.

## 5. Conclusions

In this retrospective adult orthodontic sample, the study-specific Cl-Na/Cl-Ba cranial-base angle was associated with both segmental cervical inclination and sagittal spatial coordinates. The largest associations involved the vertical coordinates of V, C2, and C3, and the magnitude of the associations decreased toward C5. These findings support the use of angular and spatial variables as complementary descriptors of segmental cervical organization.

No significant time × morphocranial-pattern interaction was observed. Thus, the longitudinal model provided no evidence of differential longitudinal changes in cervical inclination among the morphocranial groups over the observed treatment interval. This finding does not establish that orthodontic treatment has no effect on cervical posture, because the retrospective design and the absence of an untreated comparison group do not permit treatment-specific causal inference.

Clinically, segment-specific spatial and angular variables may help describe cranio-cervical variation on lateral cephalograms. Their use as diagnostic thresholds, treatment targets, or indicators of a causal cranio-cervical mechanism requires external validation, three-dimensional assessment, and prospective controlled studies.

## Figures and Tables

**Table 1:** Cephalometric reference points.

Craniofacial Region		
Cl	Clival point (Cl)	Operational posterosuperior point on the clival contour used in the study-specific tracing protocol; this point is distinct from sella (S) and should not be interpreted as the conventional dorsum sellae (DS) landmark.
Na	Nasion	Most anterior point of the frontonasal suture; represents the anterior limit of the skull base.
Ba	Basion	Posteriormost point of the occipital clivus; represents the posterior limit of the skull base.
O	Occipital base	Intersection of the tangent to the occipital base passing through ENP
ENP	Posterior nasal spine	Posteriormost point of the interpalatal suture, representing the posterior limit of the hard palate
Pt	Pterygoid point	Highest posterior point of the pterygomaxillary fossa
**Cervical Region**		
V	Vertex position of C2	Most superior point of the odontoid
Cv2ip	Position of C2	Most posteroinferior point of the body of the second cervical vertebra
Cv3ip	Position of C3	Most posteroinferior point of the body of the third cervical vertebra
Cv4ip	Position of C4	Most posteroinferior point of the body of the fourth cervical vertebra
Cv5ip	Position of C5	Most posteroinferior point of the body of the fifth cervical vertebra

**Table 2:** Cephalometric lines.

Cl-N	Anterior Cranial Base	Clivus line to Nasion
Cl-Ba	Posterior Cranial Base	Clivus to Basion line
McG	McGregor Plane	Line from ENP to the base of the occipital
Vertical	Vertical Reference Plane	Line perpendicular to the MGP plane passing through the point Pt

**Table 3:** List of study variables.

Cranial Morphology		
Cl-Na/Cl-Ba	Craniofacial pattern	Angle formed by Cl-Na and Cl-Ba
**Cervical region**		
Vector V	Position of C2	Horizontal and vertical distances from the odontoid vertex to the vertical reference line and McGregor plane, respectively.
Cv2ip Vector	Position of C2	Horizontal and vertical distances from Cv2ip to the vertical reference line and McGregor plane, respectively.
Cv3ip Vector	Position of C3	Horizontal and vertical distances from Cv3ip to the vertical reference line and McGregor plane, respectively.
Cv4ip Vector	Position of C4	Horizontal and vertical distances from Cv4ip to the vertical reference line and McGregor plane, respectively.
Cv5ip Vector	Position of C5	Horizontal and vertical distances from Cv5ip to the vertical reference line and McGregor plane, respectively.

**Table 4:** Sample distribution according to morphocranial pattern, sex, and cranial morphology.

Morphocranial Pattern	*n*	Female	Male	Age Group (Mean ± SD)	Cranial Morphology (Mean ± SD)
Brachycephalic	60	32	28	1.62 ± 0.67	111.0 ± 11.5
Mesocephalic	175	116	59	1.65 ± 0.71	120.0 ± 2.36
Dolichocephalic	62	46	16	1.84 ± 0.77	127.0 ± 2.24

**Table 5:** Spearman correlations between the continuous Cl-Na/Cl-Ba cranial-base angle and cervical variables.

Variable		rho	*p*-Value
Inclination	C2	−0.323	<0.001
	C3	−0.316	<0.001
	C4	−0.333	<0.001
	C5	−0.213	0.002
Spatial coordinates	V X	0.433	<0.001
	V Y	−0.641	<0.001
	C2 X	0.501	<0.001
	C2 Y	−0.653	<0.001
	C3 X	0.465	<0.001
	C3 Y	−0.617	<0.001
	C4 X	0.419	<0.001
	C4 Y	−0.583	<0.001
	C5 X	0.408	<0.001
	C5 Y	−0.522	<0.001

Note: C5-related analyses were based on 218 patients with an identifiable C5 landmark in both the pre-treatment and post-treatment radiographs.

**Table 6:** Descriptive statistics of segmental cervical inclination according to morphocranial pattern.

Variable	Brachycephalic	Mesocephalic	Dolichocephalic	*p*-Value
C2 inclination (°)	78.6 ± 8.21	74.9 ± 7.70	70.5 ± 8.64	<0.001
C3 inclination (°)	78.8 ± 7.83	75.1 ± 7.67	70.9 ± 8.52	<0.001
C4 inclination (°)	78.0 ± 7.66	74.2 ± 7.59	69.7 ± 8.12	<0.001
C5 inclination (°)	74.7 ± 8.60	69.1 ± 9.23	67.7 ± 8.86	0.002

Note: C5-related analyses were based on 218 patients with an identifiable C5 landmark in both the pre-treatment and post-treatment radiographs.

**Table 7:** Kruskal–Wallis analyses and effect sizes for angular and spatial cervical variables.

Variable	H	*p*-Value	η^2^(H)/ε^2^	Magnitude
**Inclination**				
C2	28.6	<0.001	0.091	Moderate
C3	27.3	<0.001	0.086	Moderate
C4	29.9	<0.001	0.095	Moderate
C5	12.2	0.002	0.050	Small
**Position**				
V X	51.6	<0.001	0.169	Large
V Y	84.0	<0.001	0.281	Large
C2 X	70.1	<0.001	0.231	Large
C2 Y	86.1	<0.001	0.287	Large
C3 X	60.8	<0.001	0.201	Large
C3 Y	74.1	<0.001	0.245	Large
C4 X	49.1	<0.001	0.160	Large
C4 Y	65.0	<0.001	0.214	Large
C5 X	34.0	<0.001	0.158	Large
C5 Y	37.8	<0.001	0.167	Large

Note: C5-related analyses were based on 218 patients with an identifiable C5 landmark in both the pre-treatment and post-treatment radiographs.

**Table 8:** Dunn–Holm post hoc comparisons between morphocranial groups.

Variable	Comparison	Adjusted *p*-Value
C2 inclination	Brachycephalic vs. Mesocephalic	0.001
	Brachycephalic vs. Dolichocephalic	<0.001
	Mesocephalic vs. Dolichocephalic	0.002
C3 inclination	Brachycephalic vs. Mesocephalic	0.002
	Brachycephalic vs. Dolichocephalic	<0.001
	Mesocephalic vs. Dolichocephalic	0.002
C4 inclination	Brachycephalic vs. Mesocephalic	0.001
	Brachycephalic vs. Dolichocephalic	<0.001
	Mesocephalic vs. Dolichocephalic	0.001
C5 inclination	Brachycephalic vs. Mesocephalic	0.005
	Brachycephalic vs. Dolichocephalic	0.003
	Mesocephalic vs. Dolichocephalic	0.362
C2 Y	Brachycephalic vs. Mesocephalic	<0.001
	Brachycephalic vs. Dolichocephalic	<0.001
	Mesocephalic vs. Dolichocephalic	<0.001
C3 Y	Brachycephalic vs. Mesocephalic	<0.001
	Brachycephalic vs. Dolichocephalic	<0.001
	Mesocephalic vs. Dolichocephalic	<0.001
C4 Y	Brachycephalic vs. Mesocephalic	<0.001
	Brachycephalic vs. Dolichocephalic	<0.001
	Mesocephalic vs. Dolichocephalic	<0.001
C5 Y	Brachycephalic vs. Mesocephalic	<0.001
	Brachycephalic vs. Dolichocephalic	<0.001
	Mesocephalic vs. Dolichocephalic	0.001

Note: C5-related analyses were based on 218 patients with an identifiable C5 landmark in both the pre-treatment and post-treatment radiographs.

**Table 9:** Descriptive statistics of cervical spatial coordinates according to morphocranial pattern.

Variable	Brachycephalic	Mesocephalic	Dolichocephalic	*p*-Value
V X	39.0 ± 3.30	41.6 ± 3.96	44.6 ± 4.26	<0.001
V Y	8.2 ± 3.8	4.6 ± 4.1	1.2 ± 4.5	<0.001
C2 X	45.8 ± 5.67	50.2 ± 5.46	55.5 ± 6.10	<0.001
C2 Y	32.8 ± 4.95	27.3 ± 5.52	22.4 ± 5.48	<0.001
C3 X	48.9 ± 7.49	54.2 ± 6.82	60.4 ± 7.79	<0.001
C3 Y	48.8 ± 5.99	42.8 ± 6.13	37.5 ± 6.64	<0.001
C4 X	52.8 ± 9.17	59.2 ± 8.37	65.4 ± 9.55	<0.001
C4 Y	64.3 ± 7.42	57.5 ± 7.57	51.7 ± 7.86	<0.001
C5 X	56.0 ± 10.3	65.5 ± 9.60	71.2 ± 10.4	<0.001
C5 Y	79.1 ± 9.1	72.6 ± 9.4	66.8 ± 9.8	<0.001

Note: C5-related analyses were based on 218 patients with an identifiable C5 landmark in both the pre-treatment and post-treatment radiographs.

**Table 10:** Correlations between V Y and vertical cervical coordinates.

Variable	rho	*p*-Value
C2 Y	0.800	<0.001
C3 Y	0.710	<0.001
C4 Y	0.638	<0.001
C5 Y	0.571	<0.001

Note: C5-related analyses were based on 218 patients with an identifiable C5 landmark in both the pre-treatment and post-treatment radiographs.

**Table 11:** Summary of principal findings, supported interpretation, and cautions.

Finding	Observed Result	Interpretation Supported by the Data	Caution
Segmental inclination	Inverse correlations across C2–C5	The study-specific cranial-base angle was associated with segmental angular variables.	Correlation does not establish direction or mechanism.
Spatial coordinates	Largest associations for V Y, C2 Y, and C3 Y	Spatial location provided information complementary to segmental inclination.	Two-dimensional coordinates do not represent full three-dimensional alignment.
Cranio-caudal pattern	Association magnitude decreased from C2 to C5	Upper cervical levels showed the largest measured associations.	C5 was not visible in all radiographs.
Longitudinal analysis	Significant time effect; no time × group interaction	The model provided no evidence of differential longitudinal changes in cervical inclination among groups during the observed interval.	No untreated control group; treatment effects cannot be isolated.

## Data Availability

The data supporting the findings of this study are available from the corresponding author upon reasonable request.
